# Association between hippocampal subfield volume estimated from ultra‐high field scans and cognitive and clinical symptoms in patients with late‐life depression and healthy controls

**DOI:** 10.1002/alz.092212

**Published:** 2025-01-09

**Authors:** Xiaochen Hu, Philip Zeyen, Gérard N Bischof, David Elmenhorst, Gregory Andrassy, Beyza Aktas, Neele Schruttke, You Na Lee, Niloofar Mokhtari, David Berron, Kathrin Giehl, Oezguer A. Onur, Rüdiger Stirnberg, Thilo van Eimeren, Alexander Drzezga, Forugh S Dafsari, Tony Stoecker, Frank Jessen

**Affiliations:** ^1^ Department of Psychiatry, University of Cologne, Medical Faculty, Cologne Germany; ^2^ German Center for Neurodegenerative Diseases (DZNE), Bonn/Cologne Germany; ^3^ University of Cologne, Faculty of Medicine and University Hospital Cologne, Department of Nuclear Medicine, Cologne Germany; ^4^ Research Center Jülich, Institute for Neuroscience and Medicine ‐ Molecular Organization of the Brain (INM‐2), Jülich Germany; ^5^ Institute of Cognitive Neurology and Dementia Research (IKND), Otto‐von‐Guericke University, Magdeburg Germany; ^6^ German Center for Neurodegenerative Diseases (DZNE), Magdeburg Germany; ^7^ Department of Neurology, University of Cologne, Medical Faculty, Cologne Germany; ^8^ Research Center Jülich, Institute of Neuroscience and Medicine (INM‐3), Cognitive Neuroscience, Jülich Germany; ^9^ German Center for Neurodegenerative Diseases (DZNE), Bonn Germany; ^10^ Excellence Cluster on Cellular Stress Responses in Aging‐Associated Diseases (CECAD), University of Cologne, Cologne Germany

## Abstract

**Background:**

Late‐life depression (LLD) is a risk factor for Alzheimer's disease (AD) dementia. Previous morphological studies have often associated LLD with atrophy within the medial temporal lobe (MTL), including the hippocampus. A number of previous studies have demonstrated the changes in several MTL subfields in LLD, such as the perirhinal cortex (PrC), cornu ammonis (CA), dentate gyrus (DG), subiculum and entorhinal cortex (EC), but with inconsistent results, which may be explained by the relatively low image resolution of the 3T scanner used in the previous studies.

**Method:**

A total of 93 individuals over the age of 60 were included in this study, of which 23 LLD patients and 29 normal controls without a history of depression underwent T1 and T2tse scans on a 7‐Tesla MRI scanner. Images were pre‐processed and roughly segmented into CA1‐3, DG, SUB, EC, and PrC (area 35, 36) using the Automated Segmentation Hippocampal Subfields (ASHS) and further separated into anterior and posterior (head and body). All segmented images were manually edited. Group comparisons of MTL subfield volumes were made, adjusting for age, sex, and years of education. Cognitive and clinical scores were correlated with the volume of each subfield.

**Result:**

LLD and controls did not differ in total hippocampal volume. LLD showed reduced volume in the head portion of the right DG (p=0.05) and a trend towards reduced ratio between left and right EC (p=0.07). No other group differences were observed. Correlational analyses revealed a significant association between bilateral hippocampal volume and TMT‐A speed, as well as the anxiety subscale of the Geriatric Depression Scale (GDS). Subfield analyses revealed significant associations between the anxiety subscale of the GDS and bilateral DG head volume (left: r=‐0.36, p=0.006; right: r=‐0.42, p=0.002) and between the left CA1 body and the cognition subscale of the GDS (r=0.29, p=0.03).

**Conclusion:**

Using ultra‐high field MRI, we demonstrated an anterior‐posterior differentiation along the hippocampal long axis in the involvement of cognitive and clinical symptoms in LLD. Future work should investigate the relationship between AD pathological changes and behavioral symptoms in LLD and whether MTL subfields may play a mediating role.

